# Electrochemical synthesis of ZnO branched submicrorods on carbon fibers and their feasibility for environmental applications

**DOI:** 10.1186/1556-276X-8-262

**Published:** 2013-06-03

**Authors:** Yeong Hwan Ko, Dandu Kamakshigari Venkata Ramana, Jae Su Yu

**Affiliations:** 1 Department of Electronics and Radio Engineering, Kyung Hee University, Yongin 446-701, South Korea

**Keywords:** Hierarchical submicrorods, Zinc oxide, Carbon fibers, Heavy metal removal, Electrochemical deposition

## Abstract

We investigated the structural and optical properties of the hierarchically integrated zinc oxide (ZnO) branched submicrorods on carbon fibers (ZOCF) by scanning/transmission electron microscopy, X-ray diffraction, and photoluminescence (PL) measurements. The ZnO submicrorods were facilely synthesized by an electrochemical deposition method on polyacrylonitrile-based carbon fiber sheets used as a substrate. After coating the ZnO seed layer on the surface of the carbon fibers, ZnO submicrorods were densely grown on the nuclei sites of the seed layer. The prepared ZOCF samples exhibited high crystallinity and good PL properties. A feasibility for environmental application in Pb(II) removal from aqueous solutions was also studied. The ZOCF adsorbent exhibited an excellent maximum adsorption capacity of 245.07 mg g^−1^, which could be practically used in Pb(II) removal from water. These fabricated ZOCFs are potentially useful for multifunctional and environmental devices.

## Background

Over the past years, ZnO nano- or microstructures have attracted great interest in a wide range of application fields such as electronic, photonic, photovoltaic, piezoelectric, and chemical sensing devices due to their unique properties [1-5]. Recently, many efforts have been made to synthesize and integrate such ZnO nanostructures on specific substrates based on functional materials including graphene, paper fibers, and conductive fabric as well as flexible or foldable plastic substrates with less weight and cost-effective productivity because their physical and chemical properties can be improved [6-9]. Synthetic strategies, e.g., hydrothermal synthesis, sol–gel method, electrochemical deposition (ED), chemical vapor deposition, and laser ablation technique, have been developed to fabricate high-purity and high-crystallinity ZnO nanostructures on functional substrates. Among them, particularly, the ED method has many advantages in producing ZnO nanostructures [10-12]. For instance, ZnO nanostructures could be grown at low temperature (75°C to 85°C) for short preparation time utilizing the ED process. Furthermore, the shape and size of ZnO nanostructures were readily tuned by controlling the external cathodic voltage and concentration of growth solution. For this reason, it would be desirable to integrate ZnO submicron structures on carbon fibers by the ED method.

Meanwhile, carbon-based materials like carbon nanotubes and nanosized metal oxides (NMOs) including TiO_2_, Fe_3_O_4_, MnFe_2_O_4_, and ZnO have been promising in the removal of various toxic metals such as Cr(VI), Cd(II), Pb(II), and Hg(II) ions due to the larger surface area and higher porosity of nanostructured materials compared to bulk materials, allowing for efficient adsorption of heavy metal ions [13-16]. Although numerous methods were already practically used for heavy metal removal from aqueous solutions, adsorption techniques have come to the forefront and are effective and economical [17]. However, NMOs are poor in mechanical strength and unfeasible in flow-through system. On the contrary, ZnO branched submicrorods on carbon fibers (ZOCF) can be employed as a complex adsorbent with the desired mechanical strength by using NMOs as host resources in permeable supports [18]. Moreover, ZnO has been considered as a promising material because of its morphological variety with nontoxic property. It is very interesting to study the removal of Pb(II) by hierarchical ZnO structures. In this work, we prepared hierarchically integrated ZnO branched submicrorods on ZnO seed-coated carbon fibers by a simple ED method and investigated their structural and optical properties. An environmental feasibility of using ZOCF for the removal of Pb(II) metals was tested.

## Methods

**Figure 1:**
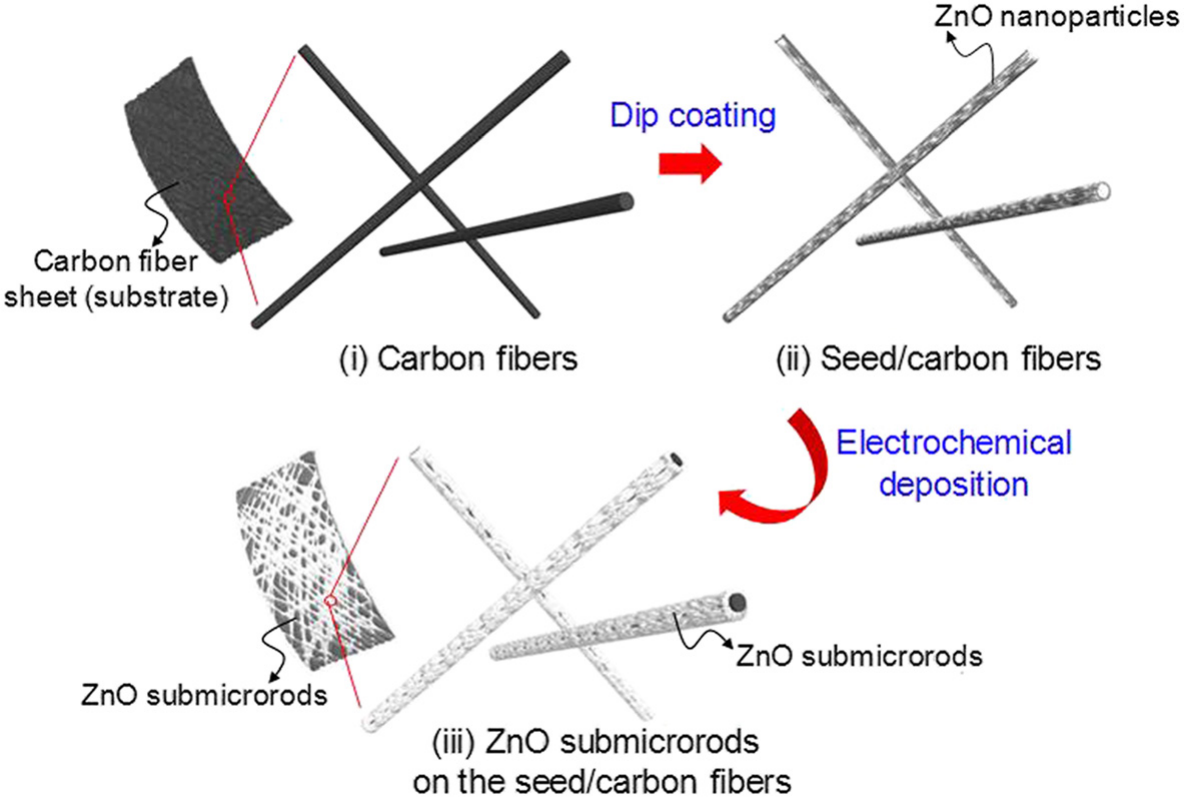
**Schematic diagram of the ZOCF fabrication procedure.** (i) Preparation of the carbon fiber substrate, (ii) the ZnO seed-coated carbon fiber substrate (i.e., seed/carbon fiber), and (iii) the ZnO submicrorods on the seed/carbon fiber.

All chemicals, which were of analytical grade, were purchased from Sigma-Aldrich (St. Louis, MO, USA) and used without further purification. The ZOCF fabrication procedure is shown in Figure 1: (i) the preparation of carbon fiber substrate, (ii) the ZnO seed-coated carbon fiber substrate (i.e., seed/carbon fiber), and (iii) the ZnO submicrorods on the seed/carbon fibers (i.e., ZOCF). The ZOCF was prepared by a simple ED process at low temperature. The ED method was carried out with a two-electrode system in which the platinum mesh/working sample acted as the cathodic electrode/anodic electrode, respectively. Practically, such simple method may be useful and reliable for synthesizing metal oxide nanostructures [19,20]. In this experiment, the industrially available carbon fiber sheet, which was made from carbonized polyacrylonitrile (PAN) microfibers by a heat treatment, was chosen as a substrate. To prepare the substrate, carbon fiber sheets of 2 × 3 cm^2^ were cleaned by rinsing with ethanol and deionized (DI) water in an ultrasonic bath at 60°C. After air drying at room temperature for 1 h, the sample was immersed into the ZnO seed solution and pulled up carefully. Here, the seed solution was prepared by dissolving 10 mM of zinc acetate dehydrate and 1 mL of sodium dodecyl sulfate solution in 50 mL of ethanol. For good adhesion, the sample was heated in oven at 130°C. Meanwhile, the growth solution was prepared by mixing 10 mM of zinc nitrate hexahydrate and 10 mM of hexamethylenetetramine in 900 mL of DI water with a magnetic stirrer at 74°C to 76°C. In order to grow the ZnO submicrorods on the carbon fibers, the seed-coated sample was dipped into the aqueous growth solution, and an external cathodic voltage of −3 V was applied between two electrodes for 40 min. Then, the sample was pulled out slowly and cleaned by flowing DI water. Field-emission scanning electron microscope (SEM, LEO SUPRA 55, Carl Zeiss, Oberkochen, Germany; Genesis 2000, EDAX, Mahwah, NJ, USA) and transmission electron microscope (TEM, JEM 200CX, JEOL, Tokyo, Japan) images were used to study the morphology and shape of the as-grown ZOCF samples. The structural properties were investigated by X-ray diffraction (XRD; M18XHF-SRA, Mac Science, Yokohama, Japan), and the optical properties were analyzed by using a photoluminescence (PL) mapping system (RPM 2000, Accent Optics, Denver, CO, USA).

The removal of Pb(II) ions using ZOCF was carried out by the batch method, and the effects of various parameters such as the pH of the solution, contact time, and Pb(II) ion concentration were studied. The pH was adjusted to a desired level by adding HCl and NaOH into 50 mL of the metal solution. Then 2 × 3 cm^2^ of the ZOCF sample weighting 0.04 g was dipped into the metal solution. After that, the samples were agitated at room temperature using a shaker water bath (HB-205SW, Han Baek Scientific Company, Bucheon, Korea) at a constant rate of 180 rpm for a prescribed time to reach equilibrium. At the end of the predetermined time, the samples were taken out. The supernatant solution was carefully separated, and the concentration of Pb(II) ions was analyzed. The metal concentrations were determined by using an inductively coupled plasma spectrometer (ICP-7510, Shimadzu, Kyoto, Japan). Blank solutions (without adsorbent) were treated similarly, and the Pb(II) ion concentrations were recorded by the mass balance equation [16]*q*_*e*_ = *V*/*m*(*C*_0_ − *C*_*e*_), where *q*_*e*_ is the equilibrium adsorption capacity of Pb(II) ions (mg g^−1^) and *C*_0_ and *C*_*e*_ are the initial and equilibrium concentrations of Pb(II) ions, respectively. Here, *V* is the volume of the solution (L), and *m* is the mass of the adsorbent (g).

## Results and discussion

**Figure 2:**
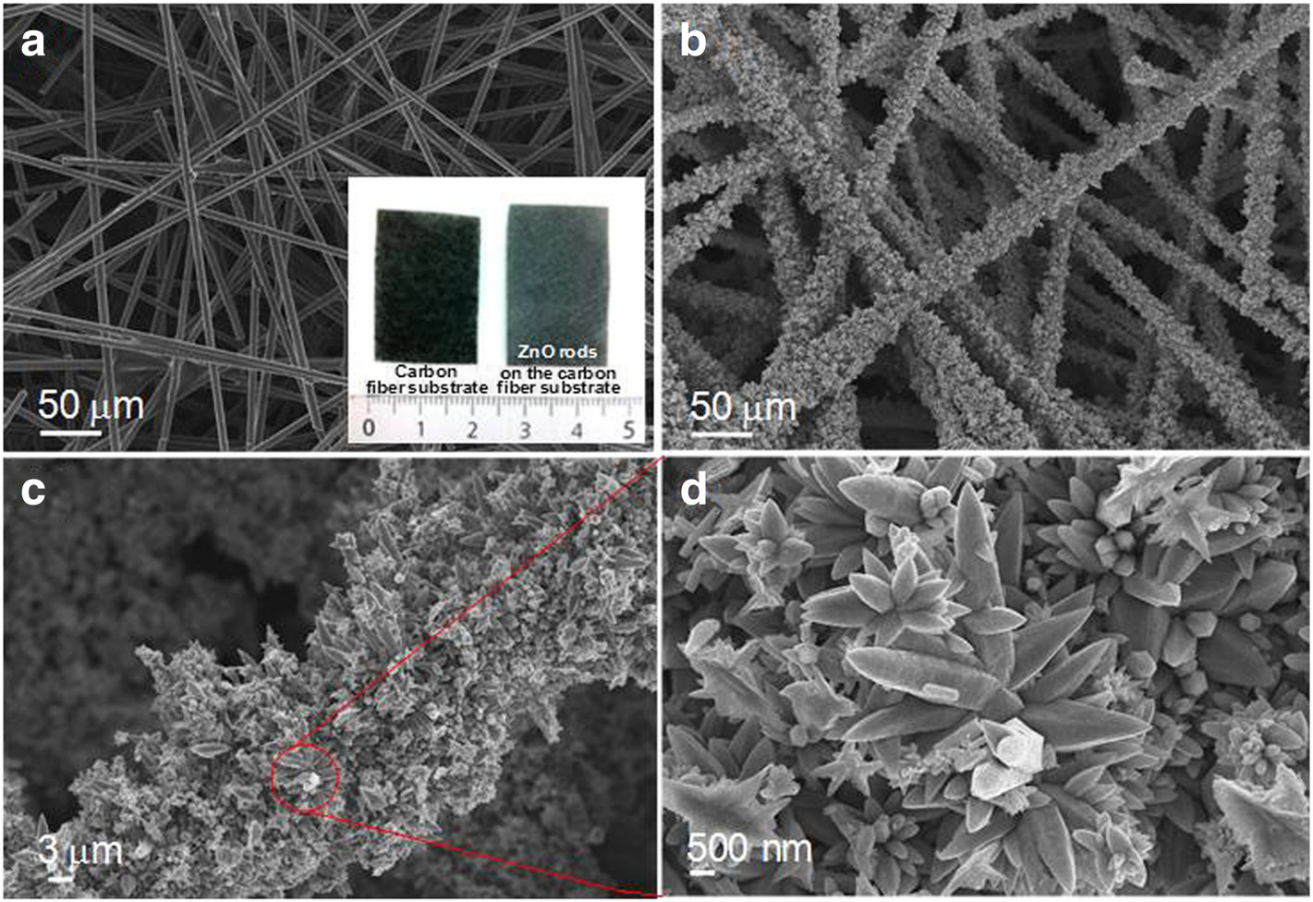
**SEM images of the samples.** SEM images of (**a**) the bare carbon fiber, (**b**) the synthesized ZnO submicrorods on the seed/carbon fiber, and (**c**, **d**) the magnified SEM images. The inset in (**a**) shows the photographic image of the carbon fiber substrates with and without ZnO submicrorods.

The SEM images of the bare carbon fiber and the synthesized ZOCF and the magnified SEM images are shown in Figure 2a,b,c,d. The inset in Figure 2a shows the photographic image of the carbon fiber substrates with and without ZnO submicrorods. As can be seen in Figure 2a, the nonwoven fabric was composed of carbon fibers with diameters of approximately 8 to 10 μm. Figure 2b shows that the ZnO submicrorods were coated over the whole surface of the carbon fibers by the process utilizing the ZnO seed layer at an external cathodic voltage of −3 V for 40 min of growth time. In addition, it could be clearly observed that the ZnO submicrorods were uniformly deposited on the carbon fiber sheet, as shown in the inset of Figure 2a. Generally, in ED process, the seed layer plays a key role because it offers nuclei sites which allow the ZnO nanostructures to grow densely [10]. The SEM images of the synthesized ZOCF with and without the seed layer were compared in the supporting information (Additional file 1: Figure S1). Furthermore, the ED process with seed layer ensured a good attachment between the synthesized ZnO and the CF substrate. As shown in the SEM images of the agitated ZOCF (Additional file 1: Figure S2), the ZnO submicrorods were well attached to the CF substrates and kept intact even after agitation at a constant rate of 180 rpm for 24 h. From the magnified SEM image in Figure 2c, somewhat complex ZnO submicrorods were densely integrated on the surface of the carbon fibers, and their sizes/heights were broadly distributed to be approximately 0.2 to 2 μm/approximately 2 to 5 μm from the microscopic observation. In the more magnified view (Figure 2d), the hierarchically structured ZnO submicrorods were aligned like a branched tree. This can be explained by the fact that the ZnO hierarchical structures are formed by subsequent growth of branches under high external cathodic voltage [12]. Indeed, these ZnO hierarchical submicrorods can be expected to provide a good adsorption capacity for heavy metal removal due to the relatively increased surface area and porosity compared to the bulk [21].

**Figure 3:**
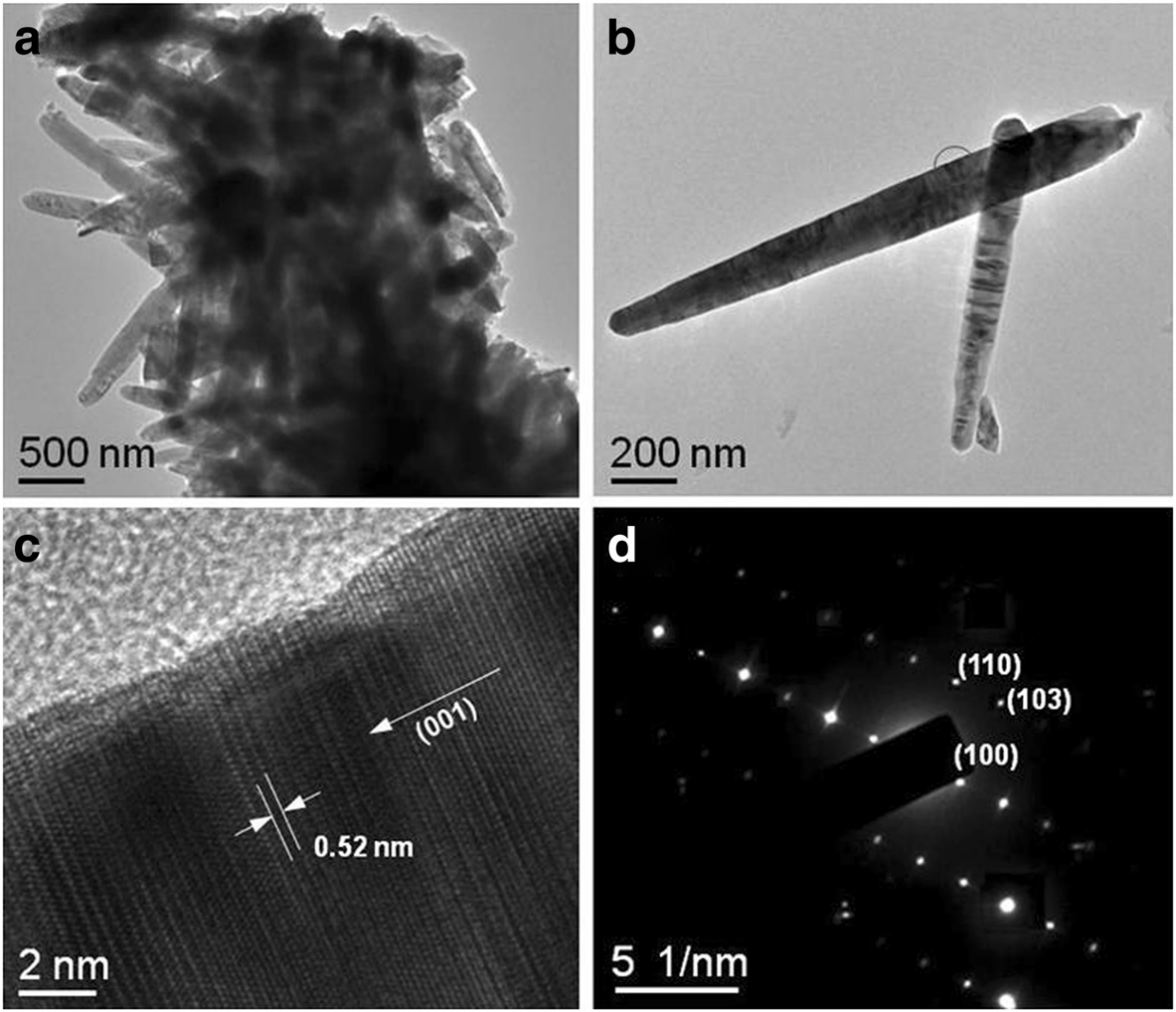
**TEM images of the samples.** TEM images of (**a**) the aggregated ZnO submicrorods and (**b**) the particular ZnO submicrorods, and the (**c**) HR-TEM image and (**d**) SAED pattern for the specific part (highlighted with a circle) in (**b**).

Figure 3a,b,c,d shows the TEM images of the aggregated ZnO submicrorods, the particular ZnO submicrorods, the high-resolution (HR)-TEM image, and selected area electron diffraction (SAED) pattern for the specific part (highlighted with a circle) in Figure 3b. To detach the ZnO submicrorods from the carbon fibers, the sample was ultrasonicated in ethanol for 1 h. As shown in Figure 3a, many ZnO submicrorods were gathered crowdedly and somewhat broken due to the ultrasonication. From the magnified TEM image in Figure 3b, the size and height of the ZnO submicrorods were estimated to be approximately 0.2 and 1.8 μm, respectively. From the HR-TEM observation (Figure 3c), the lattice fringe of the ZnO submicrorod was distinctly observed, and the distance between adjacent planes was approximately 0.52 nm, which is in good agreement with the lattice constant for the crystal plane (001) of an ideal ZnO wurtzite structure. The indexed SAED pattern confirmed that the ZnO submicrorods possessed a single crystalline hexagonal wurtzite structure.

**Figure 4:**
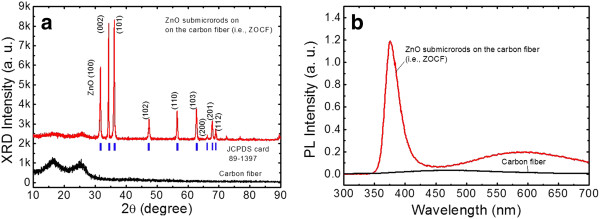
**XRD pattern and PL spectrum of the samples.** (**a**) 2*θ* scan XRD pattern and (**b**) the room-temperature PL spectrum of the CF and ZOCF.

Figure 4a,b shows the 2*θ* scan XRD pattern and the room-temperature PL spectrum of the synthesized ZOCF. For comparison, the XRD pattern and PL spectrum of the bare carbon fiber are also shown, respectively. Here, the XRD analysis and PL measurement were performed by using CuKα radiation (*λ* of approximately 1.54 Å) and a laser source (*λ* of approximately 266 nm), respectively. For the bare carbon fiber, the two broad XRD peaks were observed at 17° and 26.5° in Figure 4a, corresponding to the PAN (100) and graphite (002) planes, respectively. The crystalline graphite was formed after carbonizing the PAN by thermal treatment, but the PAN still remained [22,23]. For the synthesized ZOCF, the sharp intense XRD peaks of ZnO were clearly exhibited, and all diffraction peaks were well matched with the standard JCPDS card no. 89–1397. The dominant peaks of (002) and (101) planes were observe at 34.38° and 36.22°, respectively, indicating that the ZnO was grown perpendicularly along the c-axis and the branches were diagonally grown in the direction of the (101) plane [12,24]. As shown in Figure 4b, the ZOCF exhibited PL emission in the ultraviolet (UV) and visible regions, while the carbon fibers exhibited no PL emission. The UV emission peak in the PL spectrum was observed at 375.2 nm, corresponding to the near-band-edge emission (NBE) of ZnO with the radial recombination of free excitons. The low intensity and broad visible PL emission were caused by the deep defect level emission (DLE) of charged oxygen vacancy. The high intensity ratio of the NBE to DLE confirms that the synthesized ZnO submicrorods have a good optical property.

**Figure 5:**
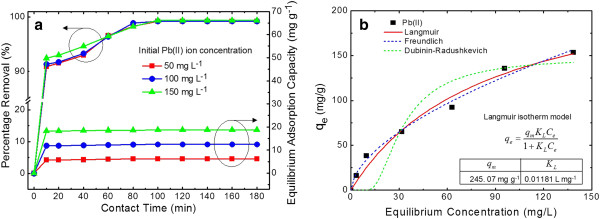
**An environmental feasibility of the sample for the removal of Pb(II) metals.** (**a**) Percentage removal and equilibrium adsorption capacity of Pb(II) onto the ZOCF adsorbent as a function of contact time at the initial Pb(II) ion concentrations of 50, 100, and 150 mg L^−1^, at pH 5.5, in the contact time range of 10 to 180 min at room temperature (25 ± 1°C) with a fixed adsorbent dose, and (**b**) the equilibrium adsorption capacity of Pb(II) ions onto the ZOCF adsorbent as a function of equilibrium Pb(II) ion concentration with nonlinear curve fits of the Langmuir, Freundlich, and Dubinin-Radushkevich isotherm models.

For a feasibility test in environmental applications, the percentage removal and equilibrium adsorption capacity (*q*_*e*_) of Pb(II) onto the ZOCF adsorbent was measured as a function of contact time at initial Pb(II) ion concentrations of 50, 100, and 150 mg L^−1^, at pH 5.5, in the contact time range of 10 to 180 min at room temperature (25 ± 1°C) with a fixed adsorbent dose, as shown in Figure 5a. The optimum pH value was determined to be 5.5 in the supporting information (Additional file 1: Figure S3). When the pH was changed from 2.0 to 9.0 to remove Pb(II) ions at the initial Pb(II) ion concentration of 50 mg L^−1^, the maximum percentage removal reached 99.58% at pH 5.5. As shown in Figure 5a, the percentage removal was dramatically increased to 90.87%, 91.36%, and 92.44% in the first step within 10 min at the initial Pb(II) ion concentrations of 50, 100, and 150 mg L^−1^, respectively, due to the increased number of active metal-binding sites on the adsorbent surface. In the second stage between 10 and 100 min, the percentage removal gradually increased because the ZOCF adsorbent was quantitatively insignificant after the first step consumption in the removal of Pb(II) ions. Above 100 min of contact time, the removal was very slow and saturated because of the repulsions between the Pb(II) ions on the adsorbate and the aqueous phases [25], finally indicating the percentage removal up to 99.2% to 99.3%. The *q*_*e*_ values of Pb(II) ions adsorbed on the ZOCF adsorbent are 6.19, 12.45, and 18.71 mg g^−1^ at 50, 100, and 150 mg L^−1^, respectively. Also, the analysis of adsorption kinetic is given in the supporting information (Additional file 1: Figure S4).

In order to determine the adsorption capacity of the ZOCF adsorbent, the adsorption amount of Pb(II) was measured in the Pb(II) ion concentration range of 10 to 500 mg L^−1^ at room temperature, keeping other parameters as constant, and then the maximum adsorption capacity was calculated by using the Langmuir isotherm model which is used successfully in many monolayer adsorption processes and can be given by *q*_*e*_ = (*q*_*m*_*K*_L_*C*_*e*_) / (1 + *K*_L_*C*_*e*_) [26], where *q*_*m*_ is the maximum adsorption capacity (mg g^−1^) of Pb(II) ions, and *K*_L_ is the Langmuir adsorption constant (L mg^−1^) related to the free energy of adsorption. Figure 5b shows the equilibrium adsorption capacity of Pb(II) ions onto the ZOCF adsorbent as a function of equilibrium Pb(II) ion concentration with nonlinear curve fits of the Langmuir isotherm model. Additionally, the well-known Freundlich and Dubinin-Radushkevich isotherm models were also compared, and the details are described in the supporting information (Additional file 1: Figure S4). The values of *q*_*m*_ and *K*_L_ were 245.07 mg g^−1^ and 0.01181 L mg^−1^. The Langmuir fit curves agreed with the experimental data. Interestingly, the ZOCF adsorbent exhibited a high *q*_*m*_ as compared with those reported in host-supported NMOs, which are summarized in Table 1. These results suggest that the ZOCF is a good adsorbent for the removal of Pb(II) and an alternative for the treatment of wastewaters containing heavy metals.

**Table 1:** Comparison of some host-supported NMOs for heavy metal removal

**NMOs**	**Host substrate**	**Pb(II)**	**Zn(II)**	**Cd(II)**	**Hg(II)**	**Reference**
			**(mg g**^**−1**^**)**	**(mg g**^**−1**^**)**	**(mg g**^**−1**^**)**	
MnO_2_	Crushed brick	0.030 mg g^−1^	-	-	-	[27]
MnO_2_	Sand	0.029 mg g^−1^	-	-	-	[27]
MnO_2_	Zeolite	0.35 mmol g^−1^	-	-	-	[28]
-	Diatomite	99.0 mg g^−1^	-	-	-	[29]
ZnO	Activated carbon	100%	-	-	-	[30]
CaTiO_2_	Al_2_O_3_	124 mg g^−1^	13.86	8.58	-	[31]
Fe_2_O_3_	-	218.53 mg g^−1^	-	212	344.8	[32]
Goethite	Sand	0.702 mg g^−1^	-	-	-	[33]
-	Sand	1.21 mg g^−1^	-	-	-	[34]
Fe_2_O_3_	Municipal sewage sludge	42.4 mg g^−1^	-	-	-	[35]
Fe_3_O_4_	-	-	-	-	227	[36]
ZnO	-	-	357	384	714	[16]
Fe_2_O_3_	-	176.33 mg g^−1^	16.97	-	303.0	[37]
ZnO	Carbon fiber	245.07 mg g^−1^	-	-	-	This work

## Conclusions

We successfully fabricated hierarchically integrated ZnO branched submicrorods on carbon fibers, i.e., ZOCF, by the ED method using a simple two-electrode system. With an external cathodic voltage of −3 V for 40 min of growth time, the ZnO submicrorods could be densely self-assembled on the ZnO seed-coated carbon fibers, which exhibited a high crystallinity and a good optical property. Furthermore, the ZOCF adsorbent exhibited an excellent maximum adsorption capacity of 245.07 mg g^−1^ for Pb(II) metal from water. The experimental kinetic and adsorption data could be understood by theoretical equation and isotherm modeling. These well-integrated ZnO submicrorods on carbon fibers can be useful for various electronic and chemical applications with a great environmental property.

## Competing interests

The authors declare that they have no competing interests.

## Authors’ contributions

YHK designed and analyzed the ZOCF by using measurements (SEM, TEM, XRD, and PL) and analyzing each sample. DKVR characterized the capacity of the sample to remove Pb(II) metals. The overall experiment and preparation of the manuscript were carried out under the instruction of JSY. All authors read and approved the final manuscript.

## Supplementary Material

Additional file 1 Additional data on the synthesis and properties of ZOCF. Click here for file
