# Supplementary material for: Electrochemical synthesis of ZnO branched submicrorods on carbon fibers and their feasibility for environmental applications
Source: Nanoscale Res Lett. 2013 Jun 3;8(1):262. doi: 10.1186/1556-276X-8-262 (PMC3689163; doi:10.1186/1556-276X-8-262)
Supplement: Additional file 1 — Additional data on the synthesis and properties of ZOCF. [file 1556-276X-8-262-S1.docx]

**Supplementary Information**

1. **Effect of seed layer**


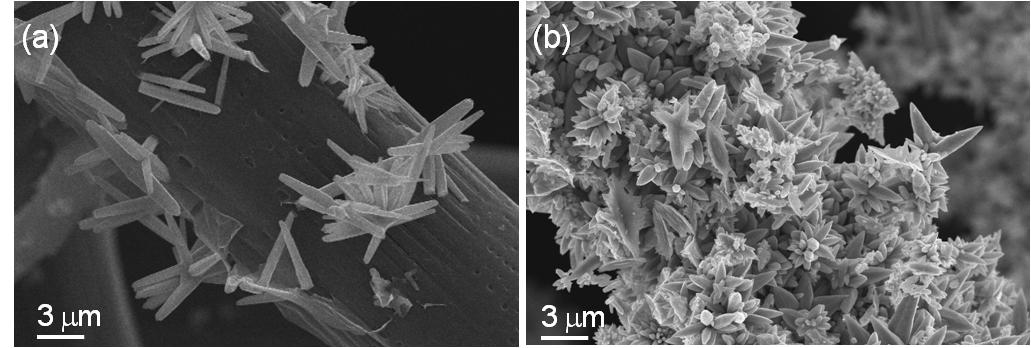


**Figure S1.** SEM images of the synthesized ZnO on carbon fibers (a) without ZnO seed layer and (b) with ZnO seed layer by the electrochemical deposition (ED) method. The zinc nitrate concentration was 10 mM and the growth temperature was 74-76 ºC. In the ED process, the external cathodic voltage of -3 V was applied between two electrodes for 40 min.

1. **Attachment test of ZnO branched submicrorods on the carbon fibers (ZOCF)**

**Figure S2.** (a) Perspective and (b) magnified SEM images of the ZOCF after agitation with a constant rate of 180 rpm for 24 h using a shaker water bath at room temperature.

1. **Effect of pH**

The effect of pH in the solution on the adsorption of Pb(II) ions onto the ZOCF adsorbent was measured in the range of 2.0-9.0 at the initial Pb(II) ion concentration of 50 mg L^-1^ as shown in Fig. S3. This can be explained in terms of pH_pzc_ (i.e., point of zero charge) and surface site distribution of the materials. The pH_pzc_ of the adsorbent was found to be 7.32 by the method described by Babic et al.^1^ When the pH_pzc_ is larger than the pH, the surface charge of the adsorbent is positive and the adsorption of metal on the surface of adsorbent may be hindered due to the charge repulsion. When the pH_pzc_ is less than the pH, the surface of the adsorbent is negatively charged, thereby it is easy for the positively charged metal to be adsorbed on the negatively charged adsorbent surface.^2^ The pH_pzc_ of the adsorbent becomes zero or less positive with the increase in the pH of the solution, hence the fraction of metal adsorption increases with increasing the pH.^3^ Initially, at pH 2.0, the percentage removal of Pb(II) was observed to be 96% and above. With increasing the pH, the maximum percentage removal reached to 99.58% at pH 5.5 (This may be ascribed to the amphoteric nature of ZnO). Therefore, further experimental work was performed at pH of 5.5 for the ZOCF adsorbent to avoid precipitation.^3^ The decrease in percentage removal after the optimum value reflects a reduction of negative surface charge density of the ZOCF.

**Figure S3.** Percentage removal of Pb(II) ions onto the ZOCF adsorbent as a function of pH at the initial Pb(II) ion concentration of 50 mg L^-1^.

1. **Adsorption kinetic**

In order to investigate the adsorption rate law of Pb(II) adsorption, the kinetic data obtained from batch experiments have been analyzed using the pseudo-first-order, pseudo-second-order and weber-Morris models. The pseudo-first-order equation of Lagergren is generally expressed by^4^

$\frac{dq_{e}}{dt}=k_{1}(q_{e}-q_{t})$. (1)

By integrating, the linear form of the above equation is followed as:

$\log(q_{e}-q_{t})=\frac{\log q_{e}-k_{1}t}{2.303}$, (2)

where *q_e_* is the equilibrium adsorption capacity of Pb(II) ions (i.e., *t*→∞), *q_t_* (mg g^-1^) is the adsorption capacity of Pb(II) ions at time *t*, and *k_1_* (min^-1^) is the pseudo-first-order rate constant of the adsorption process. Linear plots of log(*q_e_*-*q_t_*) versus t were used to evaluate the data and to determine the rate constant and *q_e_* from the slope and intercept, respectively. The linear form of pseudo-second-order kinetic rate equation proposed by Ho and Mckay is given by^5^

$\frac{t}{q_{t}}=\frac{1}{k_{2}q_{e}^{2}}+\frac{t}{q_{e}}$, (3)

where *k_2_* (g mg^-1^ min^-1^) is the pseudo–second–order rate constant of adsorption process. From the plots of *t*/*q_t_* versus *t* at the initial Pb(II) ion concentrations (Fig. S4), the *q_e_* and *k_2_* values were evaluated. The intraparticle-diffusion model (Weber-Morris) equation is represented by^6^

$q_{t}=k_{id}t^{1/2}+C$, (4)

where *C* (mg g^-1^) is the intercept and *k_id_* (mg g^-1^ min^-1/2^) is the intraparticle diffusion rate constant.

The pseudo-first-order rate constant, *k_1_*, correlation coefficient (*R^2^*), *q_e,exp_* (experimental), *q_e,cal_* (calculated) and *k_id_* (Weber-Morris) values are shown in Table S1. The *q_e,cal_* values are much lower than the corresponding *q_e,exp_*, indicating that the adsorption process has not fully followed the pseudo-first-order adsorption rate expression. Furthermore, the lower correlation factors obtained from pseudo-first–order kinetic model (0.8605-0.9385) compared to that obtained from the pseudo-second-order kinetic model (0.999-1.000) for the adsorption of Pb(II) ions indicate that the pseudo-second-order kinetic model better represents the adsorption kinetics and thus supports the assumption behind the model.

| **Pseudo-second-order kinetic** | | | **Weber-Morris** | | |
| --- | --- | --- | --- | --- | --- |
| q_e,cal_  (mg g^-1^) | k_2_(mg g^-1^ min^-1^) | R^2^ | q_e,cal_  (mg g^-1^) | k_id_  (mg g^-1^ min^-1/2^) | R^2^ |
| 6.25 | 11.4 × 10^–2^ | 0.999 | 9.88 | 0.8604 | 0.4288 |
| 12.5 | 2.86 × 10^–2^ | 1.000 | 6.42 | 0.5842 | 0.4499 |
| 18.86 | 1.25 × 10^–2^ | 1.000 | 3.00 | 0.3053 | 0.5048 |

**Figure S4.** *t*/q_t_ as a function of contact time at the initial Pb(II) ion concentrations by the pseudo-second-order kinetic model.

| Initial Pb(II) concentration (mg L^–1^) | Experimental  value  q_e,exp_ (mg g^–1^) | **Pseudo-first-order kinetic** | | |
| --- | --- | --- | --- | --- |
|  |  | q_e,cal_  (mg g^–1^) | k_1_  (mg g^–1^ min^–1^) | R^2^ |
| 50 | 6.19 | 1.057 | 3.17 × 10^–2^ | 0.9385 |
| 100 | 12.45 | 1.068 | 3.98 × 10^–2^ | 0.8602 |
| 150 | 18.71 | 1.057 | 3.1 × 10^–2^ | 0.9315 |

**Table S1.** Rate constants and equilibrium parameters of pseudo-first-order kinetic, pseudo-second-order kinetic and intraparticle diffusion models for the Pb(II) adsorption onto the ZOCF adsorbent.

1. **Equilibrium adsorption isotherm models**

Freundlich model can be applied for non-ideal adsorption on heterogeneous surfaces and multilayer adsorption. The Freundlich model is given by^7^

$q_{e}=k_{f}C_{e}^{1/n}$, (5)

where *k_f_* and *n* are the Freundlich constants related to the adsorption capacity and adsorption intensity, respectively. Dubinin-Radushkevich isotherms equation is represented as^8^

$q_{e}=q_{m}\exp(-K[RT\ln(1+\frac{1}{C_{e}})]^{2})=q_{m}\exp(-K\varepsilon^{2})$, (6)

where the *q_m_* is the maximum adsorption capacity (mg g^-1^) of Pb(II) ions, ε is the polanyi potential which is equal to *RT·*ln(1+1/*C_e_*), where *R* (8.314 × 10^–3^ kJ mol^-1^ K^-1^) and *T* (K) are the universal gas constant and the absolute temperature, respectively. The *K* (L g^-1^) is the equilibrium constant obtained by multiplying the Langmuir constant *q_m_* and *K_L_*.^9^ The Langmuir, Freundlich, and Dubinin-Radushkevich isotherm fitting constants for the Pb(II) adsorption onto the ZOCF are listed in Table S2.

**Table S2.** Langmuir, Freundlich and D–R isotherm fitting constants for the Pb(II) adsorption onto the ZOCF.

| **Model** | **Values** |
| --- | --- |
| **Langmuir** |  |
| *q_m_* (mg g^-1^) | 245.07 |
| *K_L_* (L mg^-1^) | 0.0118 |
| R^2^ | 0.9803 |
| χ^2^ | 80.86 |
| **Freundlich** |  |
| *k_f_* (mg g^-1^) | 9.09 |
| *n* | 1.73 |
| R^2^ | 0.9916 |
| χ^2^ | 34.22 |
| **Dubinin-Radushkevich** |  |
| *q_m_* (mg g^-1^) | 148.60 |
| K (L g^-1^) | 0.8082 |
| R^2^ | 0.8769 |
| χ^2^ | 506.60 |
|  |  |

The Freundlich constants, i.e., *k_f_* and *1/n* values, are 9.76 and 0.89, respectively, and the *n* value is found to be greater than 1 which is a favorable condition for adsorption.^10^ And the *R^2^* values are greater than 0.98 (for Langmuir and Freundlich isotherms compared to Dubinin-Radushkevich isotherm) and the Chi-square (χ^2^) values of two isotherms are inferior. Here, it is concluded that the adsorption of Pb(II) ions followed both the Freundlich and Langmuir isotherm models.

**References**

1. Babic BM, Milonjic SK, Polovina MJ: **Point of zero charge and intrinsic equilibrium constants of activated carbon cloth.** *Carbon* 1999, **37**:477-481.

2. Shao DD, Jiang ZQ, Wang XK: **SDBS modified XC-72 carbon for the removal of Pb(II) from aqueous solutions.** *Plasma Process Polym* 2010, **7**:552-560.

3. Sheela T, Nayaka YA, Viswanatha R, Basavanna S, Venkatesha TG: **Kinetics and thermodynamics studies on the adsorption of Zn(II), Cd(II) and Hg(II) from aqueous solution using zinc oxide nanoparticles.** *Powder Technol* 2012, **217**:163-170.

4. Lagergren S: **About the theory of so-called adsorption of soluble substances, Kungliga Svensla Vetenskapsakademien** Press, Handlinger, 1898

5. Ho YS, McKay G: **Pseudo-second order model for sorption processes.** *Process Biochem* 1999, **34**: 451-465.

6. Weber WJ, Morris JC, Sanit J: **Kinetics of adsorption on carbon from solution.** *Eng Div ASCE* 1963, **89**:31-60.

7. Freundlich H: **Adsorption in solution.** *Phys Chem Soc* 1906, **40**:1361-1368.

8. Dubinin MM, Radushkevich LV: **The equation of the characteristic curve of activated charcoal.** *Proc Acad Sci USSR* 1947, **55**:331-333

9. Aksu Z, Isoğlu IA: **Removal of copper(II) ions from aqueous solution by biosorption onto agricultural waste sugar beet pulp*.*** *Process Biochem* 2005, **40**:3031-44.

10. Chandra V, Park J, Chun Y, Lee JW, Hwang IC, Kim KS: **Water-dispersible magnetite-reduced graphene oxide composites for arsenic removal.** *ACS Nano* 2010, **4**:3979-3986.
